# Efficacy of Transcranial Magnetic Stimulation (TMS) on Cognitive and Psychiatric Symptoms in Patients with Mild Traumatic Brain Injury

**DOI:** 10.1192/j.eurpsy.2026.11745

**Published:** 2026-07-31

**Authors:** W. Aldulaimy, A. Shah

**Affiliations:** 1 Psychiatry; 2 Texas Institute for Graduate Medical Education and Research, San Antonio, United States

## Abstract

**Introduction:**

Patients with mild Traumatic Brain Injury (mTBI) often suffer from comorbid neuropsychiatric symptoms and cognitive deficits challenging to treat conventionally.

**Objectives:**

TMS is a known treatment for refractory depression, but we set out to find its effects on not only depression, but PTSD, Anxiety, and cognition, specifically memory and attention.

**Methods:**

This retrospective study of patients from Mendala PolyTrauma Clinic evaluated TMS efficacy in 13 patients with mTBI history (9 Male, 4 Female, Mean Age: 41.2 years). All patients presented with clinical complexity including Major Depressive Disorder (MDD), Post-Traumatic Stress Disorder (PTSD), and Mild Neurocognitive Disorder. Pre- and post-treatment scores were collected using PHQ-9, GAD-7, PCL-5, and a comprehensive neurocognitive battery.

**Results:**

In this clinically complex cohort of patients with mTBI, TMS was associated with significant improvements in depressive and trauma-related symptoms. Findings also show potential for TMS to ameliorate cognitive deficits in Working Memory and Episodic Memory, although effects varied across patients and domains.

The variability in response suggests that patient selection, symptom profiles, and TMS targeting strategies may be key factors in optimizing outcomes. These findings support TMS as a promising therapeutic tool for addressing the multifaceted neuropsychiatric and cognitive sequelae of TBI.

**Conclusions:**

The variability in response suggests that patient selection, symptom profiles, and TMS targeting strategies may be key factors in optimizing outcomes. These findings support TMS as a promising therapeutic tool for addressing the multifaceted neuropsychiatric and cognitive sequelae of TBI.

The Path Forward

Larger, Prospective Trials: Conduct robust, controlled studies with larger cohorts to validate these promising preliminary findings.

Optimizing Protocols: Further research into patient selection criteria, personalized TMS targeting, and treatment parameters to maximize efficacy.

Long-term Outcomes: Investigate the sustained effects of TMS on cognitive and psychiatric symptoms over extended periods.

Biomarker Identification: Explore neural biomarkers that predict treatment response to guide clinical application.

**Disclosure of Interest:**

None Declared

